# Design and implementation of real-time object detection system based on single-shoot detector and OpenCV

**DOI:** 10.3389/fpsyg.2022.1039645

**Published:** 2022-11-02

**Authors:** Fazal Wahab, Inam Ullah, Anwar Shah, Rehan Ali Khan, Ahyoung Choi, Muhammad Shahid Anwar

**Affiliations:** ^1^College of Computer Science and Technology, Northeastern University, Shenyang, China; ^2^BK21 Chungbuk Information Technology Education and Research Center, Chungbuk National University, Cheongju, South Korea; ^3^School of Computing, National University of Computer and Emerging Sciences, Faisalabad, Pakistan; ^4^Department of Electrical Engineering, University of Science and Technology, Bannu, Pakistan; ^5^Department of AI and Software, Gachon University, Seongnam, South Korea

**Keywords:** computer vision, deep learning, image recognition, object detection, object recognition, single shoot detector

## Abstract

Computer vision (CV) and human–computer interaction (HCI) are essential in many technological fields. Researchers in CV are particularly interested in real-time object detection techniques, which have a wide range of applications, including inspection systems. In this study, we design and implement real-time object detection and recognition systems using the single-shoot detector (SSD) algorithm and deep learning techniques with pre-trained models. The system can detect static and moving objects in real-time and recognize the object’s class. The primary goals of this research were to investigate and develop a real-time object detection system that employs deep learning and neural systems for real-time object detection and recognition. In addition, we evaluated the free available, pre-trained models with the SSD algorithm on various types of datasets to determine which models have high accuracy and speed when detecting an object. Moreover, the system is required to be operational on reasonable equipment. We tried and evaluated several deep learning structures and techniques during the coding procedure and developed and proposed a highly accurate and efficient object detection system. This system utilizes freely available datasets such as MS Common Objects in Context (COCO), PASCAL VOC, and Kitti. We evaluated our system’s accuracy using various metrics such as precision and recall. The proposed system achieved a high accuracy of 97% while detecting and recognizing real-time objects.

## Introduction

The computer vision (CV) field may be the best arrangement when considering its various application areas. A significant number of these applications include tasks that necessitate either working in a dangerous domain, a large handling force, access to and utilization of massive data databases or dreary schedules for individuals to complete. The conditions under which CV frameworks are used range from assembling plants to clinic careful suits. For example, CV is frequently used for quality control in the assembly of frames. The CV framework outputs fabricated items in the assembly framework application zone to distinguish imperfections and give control signals to a mechanical controller to consequently evacuate flawed parts. Frameworks to naturally analyze skin tumors and neurosurgeons during complex procedures, such as mind medical procedures, are later examples of medicinal frameworks created with CV strategies.

The process of recognizing a moving or non-stationary object in a video sequence is known as object detection. This is the initial and most crucial step in tracking moving objects. To gain a thorough understanding of images, we would not only classify them but also attempt to precisely guess the concepts and locations of objects contained in each image. Object detection (Murugan et al., 2019) is the name given to this task, which is divided into subtasks such as skeleton detection, face detection, and pedestrian detection. Object detection is a computer-challenging technology that is related to CV and image processing. It deals with identifying instances of semantic objects of a certain type (such as people or cars) in advanced pictures and recordings. Well-researched areas of object detection include confronting discovery and person-on-foot location. Most applications of object location are in numerous regions of CV, counting video observation, and picture recovery.

Object detection is one of the most difficult problems in CV, and it is the first step in a few CV applications. An object discovery framework’s goal is to identify all occurrences of objects of a known category in an image and it is a particularly difficult task in CV (Zhao et al., 2019). Somewhat obstructed object detection seeks to develop computational techniques that provide one of the most basic pieces of data required by CV applications: Where are the objects located? As one of the essential complications of CV, object detection serves as the foundation for many other CV tasks, such as instance segmentation (Mansoor et al., 2019), image captioning, object tracking, and so on.

The study of “CV,” or CV for short, aims to develop the methods that will allow computers to “see” and understand the content of computerized images such as pictures and videos. Because people, particularly children, illuminate the problem of CV insignificantly, it appears to be a simple one. It is, by the way, a generally unresolved issue due to both the limited understanding of natural vision and the complexities of vision discernment in an active and constantly changing physical world. The significance of CV lies in the issues it can shed light on. It is one of the most cutting-edge technologies, allowing communication between the developed and developing worlds. CV allows self-driving cars to understand their surroundings. Cameras in various locations around the vehicle record video and feed it to a CV program, which creates images in real-time to identify activity signs and street limits.

A traffic surveillance system can benefit from CV techniques (Mishra and Saroha, 2016). Traffic surveillance systems that identify and recognize moving objects are an important topic in CV (Biswas et al., 2019). Evaluating the sequence of frames removed from the video allows for a better understanding of how moving objects behave. It eliminates the issues associated with traditional methods that rely on human operators. Depending on the degree of manual involvement, these systems are classified as fully automatic, semi-automatic, or manual. The most important and critical component of CV applications is moving object detection (Luo et al., 2018; Runz et al., 2018). The importance of CV cannot be overstated in today’s world. Many of our critical organizations, including the Security Organization, place a high priority on CV applications.

We can clearly state from our investigation and research that several frameworks are being used to detect objects. Given that our system is not a cutting-edge innovation, nor is the entire CV field, it has been used to develop a number of related frameworks. Moreover, with CV, a part of the data is gotten from huge and small chunks of information sets and handled into important data for encouraging utilization or preparing purposes. Some of the modern object detection techniques include CNN, R-CNN, Faster R-CNN, Fast R-CNN, and YOLO (Du, 2018; Wei and Kehtarnavaz, 2019). These are the different deep learning methods that are currently used for real-time object detection (Asadi et al., 2018; Chandan et al., 2018). We can also use these techniques for other purposes such as health concerns, action detection, and so on.

The study’s main contribution is the design and implementation of real-time object detection and recognition systems using the SSD algorithm and deep learning techniques with a pre-trained model. Our proposed system can detect static and moving objects in real time and classify them. The primary goals of this study were to investigate and develop a real-time object detection system that uses deep learning and neural systems to detect and recognize objects in real time. Furthermore, we tested the free, pre-trained models with the SSD algorithm on various types of datasets to determine which models have high accuracy and speed when detecting an object. Besides this, the system must be operable on reasonable equipment. During the coding procedure, we evaluated various deep learning structures and techniques and developed and proposed a highly accurate and efficient object detection system.

The rest of the paper is structured as follows: section “Related work” represents the related work, whereas, section “System model” is the system model. Section “System design” is the system architecture, and section “Experimental results and evaluation” is the experimental results and evaluation. Finally, section “Conclusion” concluded this work.

## Related work

The significance of CV lies in the issues it can shed light on. It is a cutting-edge technology that enables the communication between developed and developing countries. Self-driving cars can understand their surroundings thanks to CV. Cameras capture video from various points around the vehicle and feed it to a CV program which then forms the images in real time to discover street limits, study activity signs, and distinguishes. A traffic surveillance system can benefit from CV techniques where the detection and recognition of non-stationary or moving objects is an important topic. Analyzing the frame sequence extracted from the live video gives us more information about how moving objects behave. It eliminates the issues associated with traditional methods that rely on human operators. Depending on the degree of manual involvement, these systems are classified as fully automatic, semi-automatic, or manual. The most important and critical component of CV applications is moving object detection (Runz et al., 2018).

Technology has advanced rapidly in the recent years. Artificial intelligence and CV are making significant strides in modern times, thanks to the development of powerful microprocessors and high-quality cameras. With the use of these technologies, CV-based real-time object detection can detect, locate, and trace an object from an image or a video. The authors propose a method for integrating this real-time system into a web page in Singh et al. (2021). The TensorFlow object detection API is used to detect objects, and live video is streamed through a password-protected login web page that can be accessed from any device. The system draws a box around the object and displays the detection accuracy. Similarly, the authors of Bian et al. (2021) and Martinez-Alpiste et al. (2022) presented a variety of approaches for CV, including OpenCV and SSD-MobileNet, object recognition, and so on.

Several recent deep learning approaches can be used to localize, classify, and detect the object. Each of these methods detects and recognizes the object using a different mechanism. In this section (Tufail et al., 2021a,b; Khan et al., 2022), we will discuss a few of them that are currently used for object detection and recognition (Mao et al., 2016; Shin et al., 2018). CNN, R-CNN, Fast R-CNN, single-shoot detector (SSD), and Faster R-CNN are the most common (Hung and Carpenter, 2017). Because the Faster R-CNN is a member of the CNN family, we will explain it in detail, as well as the R-CNN and the Fast R-CNN, and then, we will discuss the SSD, which will be used in our proposed system (Manana et al., 2018; Nalla et al., 2018). According to the study, the SSD is the fastest among the other object detection techniques (Zhou et al., 2017). (1): Faster R-CNN (region-based convolutional neural network) (Zhang and Yang, 2017; Ren et al., 2018). To prepare the shared features, faster R-CNN employs the substitute preparation mode. Its employees then plan to begin the weight of the RPN, extract the appropriate proposition from the prepared dataset, and train the Faster R-CNN show with the recommendations repeatedly until the result meets well (Risha and Kumar, 2016; Xu et al., 2017).

## System model

The primary goal of this system is to detect a static object from an image and a moving object from a video to display the object’s class. Moreover, the functional requirements describe what the system does. The main functional requirements for our proposed system include both static object recognition and moving object recognition (Yang et al., 2018). These functional requirements are the data processing module, deep learning module, static object detection module, moving object tracking module (Shilpa, 2016), pre-defined object module, and object recognition module. The proposed system takes an image from the camera, matches it with the dataset, matches it with the dataset classes, runs the pre-trained models, and finally boxes the object and displays the object instance with the accuracy level. The system modules are the functional requirements. We have a total of six modules in our system.

**FIGURE 1:**
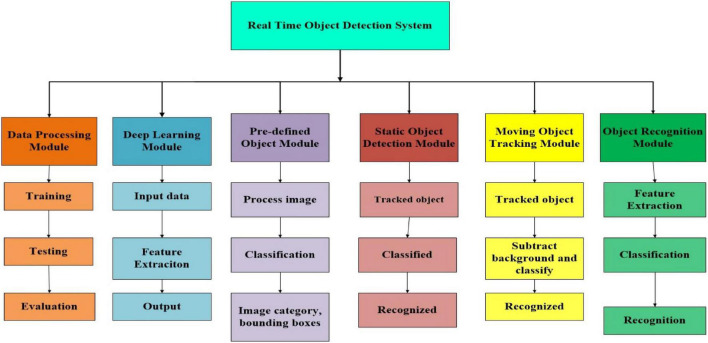
The functional requirements system modules.

Figure 1 depicts the system modules and explains the individual operations of each module. We will explain each module of the system in detail, including its figure and operating procedure, before combining these modules into the proposed system.

### Data processing module analysis

In this section, we will discuss image datasets, which are used to train and benchmark various object detection procedures. This section will go over the datasets that will be used in our real-time object detection system. There are numerous free datasets available on the internet that can be used in deep learning techniques. DNN requires a large amount of labeled data (structured data) for training the model; currently, the most used datasets for object detection are ImageNet, PASCAL VOC, and MS Common Objects in Context (COCO).

#### Kitti dataset

Kitti is a dataset composed of stereo cameras and lidar scanners in rural, urban, and highway driving settings. It is divided into 10 categories: vans, small cars, trucks, sitting people, pedestrians, cyclists, miscellaneous, and trams, and do not care (Zhao et al., 2016). The images are 1,382 × 512 in size, and 7,500 of them provide 40,000 object labels classified as easy, modest, or difficult based on how widely the images are obstructed and truncated (Salvador et al., 2016).

#### MS common objects in context dataset

MS COCO stands for common object in context. Microsoft sponsors COCO, and the annotation comprises categories, position information, and a semantic text description of the image. The COCO dataset’s open source also contributes to the advancement of object detection. Microsoft-sponsored COCO is new image segmentation, recognition, and captioning dataset. This dataset’s open-source has made significant advances in semantic segmentation in the recent years (Girshick et al., 2015; Wang et al., 2016), and it has become a “standard” dataset for image semantic understanding performance, with COCO posing a unique challenge (Chen et al., 2018).

#### PASCAL VOC dataset

PASCAL VOC gives a standard picture naming and assessment framework. The PASCAL VOC picture dataset consolidates 20 groups; the dataset highlights a high-quality and fully named image, which is especially useful for analyzing calculation execution. The PASCAL VOC (plan examination, quantifiable illustrating, and computational learning visual address classes) provides standardized image datasets for dissent lesson confirmation as well as a common set of devices for retrieving the datasets and clarifications.

#### Training and testing data

After that, the datasets are divided into train and test subsets. During the experiment, we will randomly divide our dataset in an 80:20 ratio. The Results section will explain the relationship between the training data and the detector’s presentation. A train-test split can be achieved in several ways. Even so, prediction improves if the distribution of classes on both subsets is sufficiently balanced. After dividing the dataset into two subsets, we must convert the annotated XML files to a TensorFlow-compatible file format.

**FIGURE 2:**
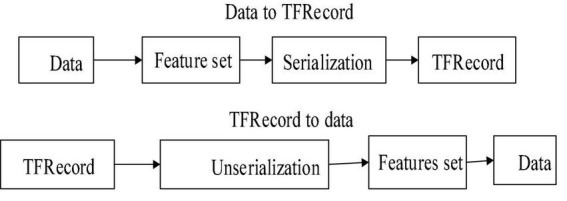
Data conversion to TF record.

TensorFlow performs the batch operation using its native file format TFrecord (record). Dissimilar to different stages which do the greater part of the clustering procedure straightforwardly from the pictures, TensorFlow uses a solitary document for the bunch activity. In the TF record, pictures are changed over into the Numpy cluster. This organization for preparing the huge dataset blends coordinate dataset and system engineering just as procedure the huge dataset that does not suitable into the retention. This configuration is the record-based twofold organization that is applied for data preparation and testing in various TensorFlow applications. There are numerous possibilities available for information pre-handling. Before converting the described dataset to TFrecord (parting XML document) or after utilizing TFrecord work, the preparation set and testing split should be possible. The XML document preparation and testing should have been done in the TFrecord record position. The data conversion to TF record is shown in Figure 2.

### Object detection deep learning module

Deep learning, a branch of machine learning and artificial intelligence, focuses on the training of computational models composed of multi-layered artificial neural networks. An ANN with multiple layers is referred to as a deep neural network (DNN). DNNs have more than two layers of hidden neurons between the input and output layers, which corresponds to the network’s depth. Modern accuracy in speech recognition, object detection, image classification, language translation, and other areas has been significantly enhanced by DNNs. Deep learning strategies depend on learning portrayals (highlights) from information, for example, content, pictures, or recordings, instead of actualizing task-explicit calculations. Learning can either be solo or regulated. Be that as it may, a large portion of the pragmatic frameworks conveys administered figuring out how to use the advantages of deep learning (Saqib et al., 2017). Managed learning, fundamentally, implies gaining from market information.

### Pre-defined objects module

**FIGURE 3:**
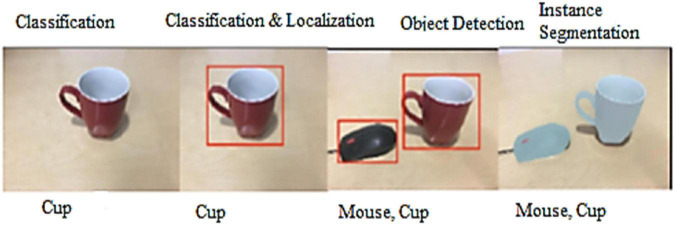
Classification, localization and instance segmentation.

Pre-defined objects mean that the object that we have already defined and labeled, it means the labeled datasets. In modern CV, object detection is now considered alongside object recognition (classification), localization, following, and data extraction from the object. Object detection is inextricably linked to these forms. The goal of classification is to determine the object’s class or identify its nature. The location of the object(s) within the image or the outline is determined by localization. The development and status of the object may be influenced through object tracking in video or live recording. The goal of the object detection framework is to classify and locate all objects displayed in a picture. The locator’s input is a picture of the object, and the output could be a list of the bounding box. The classification, localization, and instance segmentation is shown in Figure 3.

### Static object detection module

The object detection key aim is to find all instances of objects from an identified class in an image, such as people, cars, or faces. A static object is a stationary object that is not moving. The exhibition of deep learning strategies increases with an expansion in the measure of preparing information rather than customary learning techniques that soak in execution; this trademark makes the deep learning strategies versatile. Because a deep neural system is made up of many layers, it includes learning portrayals with varying degrees of complication and deliberation. As previously stated, the underlying layers become acquainted with the low-level highlights and then pass them on to the subsequent layers. The subsequent layers then accumulate elevated-level highlights based on the recently learned lower-level highlights.

1. Age: Age is a parameter that keeps track of the number of frames in which the object has not moved.
2. Type: Type primarily represents the object’s status.
3. The object can be new, matched, or occluded: Before processing the next frame, the new object is added to the previous frame.

### Moving object tracking module

In this section, we will discuss moving objects such as moving persons or cars. The detection of the physical movement of an object in each location or region is known as moving object detection. The movement of moving objects can be tracked and analyzed by acting as a division between moving objects and stationary regions or locales. Cameras distinguish moving objects around the vehicle when it is stopped or gradually maneuvering; in our framework, the camera will identify and recognize the moving protest, such as a person on the road or a car on the street. The framework then warns the driver visually and audibly in many smart frameworks. There are two frameworks: one makes use of the all-encompassing See Screen and four cameras mounted on the front, back, and sides of the vehicle, whereas the other makes use of a single camera mounted on the raise. The four-camera system can alert drivers while they are in three different motions: stopping or shifting into neutral, moving forward, and backing up. The front and rear cameras recognize moving objects independently as the vehicle moves forward or reverses.

**FIGURE 4:**
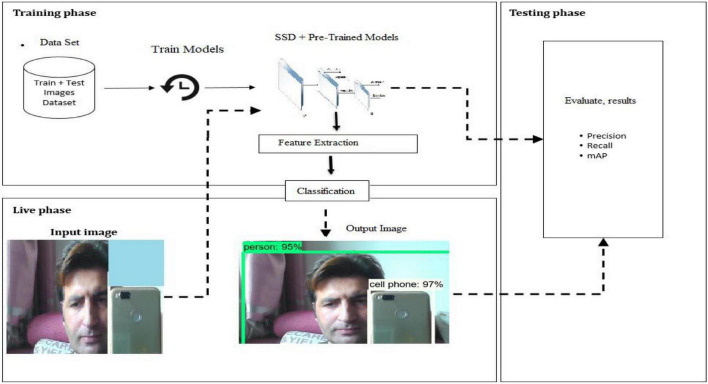
The flow chart of the deep learning module.

The framework uses a simulated bird’s eye view image to identify moving objects around the car when it is in stop or neutral. A single rear-view camera framework on a vehicle allows it to detect moving objects behind it (Manana et al., 2017; Shi et al., 2017; Zhiqiang and Jun, 2017). With the help of the cameras, the framework creates video symbolism that it uses to locate moving objects. The framework that uses the Around See Screen has been modified to analyze video signals from the four cameras that are attached to the front, rear, and both side-view mirrors of the car. Then, it can instantly distinguish moving objects around the vehicle. Depending on where the transmission is moving, it can decide which of the three options applies—moving forward, stopping, or backing up. The flow chart of the deep learning module is shown in Figure 4.

#### Background subtraction

Background subtraction is used to separate a frontal area object from its surroundings. The basic strategy for using this procedure is to create an establishment show that speaks to the scene. The establishment demonstrates capacities as a kind of the point of view and ought to in this way be reliably revived and contain no moving things. Each edge is at that point differentiated with the establishment demonstrate with the objective that alterations within the picture can be seen. By looking at each video diagram against the establishment demonstrates that it is conceivable to see moving things as distant as deviations from the reference demonstrate.

The calculations utilized for establishment subtraction are essential and clear to utilize, and the strategy is besides greatly fragile to the changes in nature. The establishment subtraction technique can be confined into two get-togethers, recursive techniques, and non-recursive frameworks. Recursive strategies base the establishment demonstrate on each video layout by recursively reviving the establishment demonstrate. The consequence of this is the model that can be influenced by info edges handled in a far-off past. Contrasted with non-recursive systems, this technique requires less memory stockpiling, yet possible blunders out of sight model can wait for a more drawn-out timeframe. Non-recursive strategies store a cushion with the keep going on video outlines.

#### Feature extractor

**FIGURE 5:**
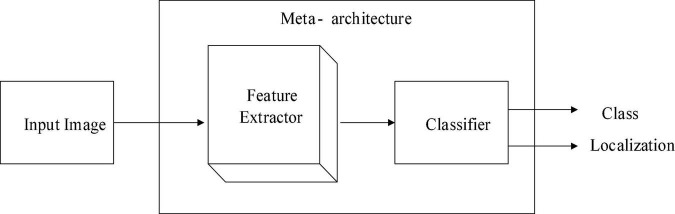
Feature’s extractor and classifier in architecture of meta data.

A crucial element of the object detection model used to extract object features from data is the feature extractor. The following figure depicts the area meta-architecture, extractor, and classifier that make up the object detection illustration structure. As shown in Figure 5, the input picture is routed through the included extractor, which separates the features from the image. The classifier then classifies the class and the area of the object within the input image using the features that were removed from the image.

The feature extractor’s deep architecture can be used to improve accuracy while reducing computational complexity. In object detection meta-structures, popular feature extractors such as AlexNet, Inception, MobileNet, NAS, ResNet, and VGG can be used. We will use a pre-trained model for feature extraction because MobileNet is more compatible with SSD.

#### Image scaling

Through pixel replication or interpolation, the image is zoomed. Scaling can be used as a low-level preprocessor in a multi-stage image processing chain that operates on scaled features, to alter the visual appearance of an image, to alter the amount of information stored in a scene representation, or for both purposes. Compressing or expanding an image along its coordinate directions is the process of scaling, since there are various methods for zooming and subsampling.

### Object recognition module

**FIGURE 6:**
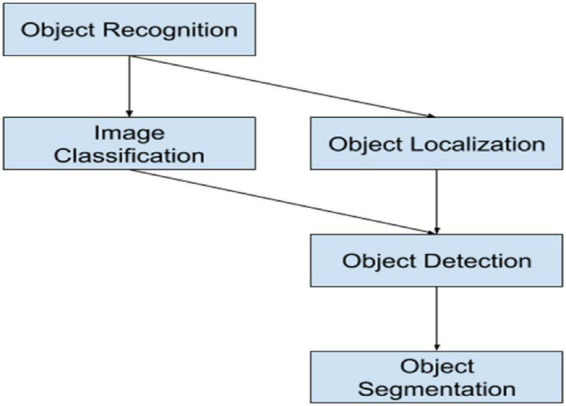
Object recognition steps.

Object recognition entails recognizing and identifying the object class. In reality, we have various object classes such as humans, cars, cups, bottles, and so on. The task at hand is to locate a specific object in an image or even a video sequence. Because, unlike humans, object recognition engines can distinguish and recognize a wide range of objects in images or recordings that may differ in the perspective, color, measure, or even when the object is slightly deterred, it may be a serious vision problem. The risk of identifying an object in an image refers to a labeling issue based on well-known object models. In essence, given a blank image containing the objects of interest and a set of names corresponding to a set of models accessible within the framework, the framework may be able to appropriately assign the names to each region within the image. Figure 6 shows how the difficult task of identifying an object in an image is defined as a labeling problem based on recognized object models. In essence, given a non-specific image containing the objects of interest and a set of names, the framework may be able to properly assign the names to the specific regions within the picture when compared to a set of models accessible within the framework.

### Models analysis

In this section, we will go over pre-trained and fine-tuned models. We will explain which models we will use in our project to create a successful real-time object detection system based on SSD.

#### Pre-trained model

A model that has been previously trained is referred to as pre-trained. A pre-trained model can be used as a starting point or for out-of-the-box inference instead of creating and training a model from scratch. Even though a pre-trained model is time-efficient, it is not always 100% accurate. Table 1 shows some pre-trained models. There are numerous pre-trained models available for free on the Tensorflow zoo website. We will need to use pre-trained SSD_MobileNet_v1_cco models, SSD_MobileNet_v2 models, and VGG16 models, which we will design in Python and implement with the SSD algorithm. We will look at which pre-trained models have high accuracy with the SSD and draw conclusions.

**TABLE 1:** Common pre-trained models.

Name	Speed (ms)	COCO mAP [∧1]	Productivity
SSD_MobileNet_v1_coco	30	21	Boxes
Faster_RCNN_Inception_v2	58	28	Boxes
Faster_RCNN_resnet_v2	89	30	Boxes
Faster_RCNN_resnet_v1	106	32	Boxes
SSD_mobilenet_v2_coco	31	22	Boxes
SSD_inception_v2_coco	42	24	Boxes

## System design

We go over the entire design process of our proposed system. We design various modules such as the data processing module, the deep learning module, and the object detection module. We explain each module with the help of figures that depict the design process.

### System architecture

A system architecture is the intangible representation of a framework’s structure, behavior, and other aspects. An architecture description is a formal depiction and representation of a system, organized in a way that promotes thinking about the framework’s structures and practices. The system architecture is essentially the overall design of the system, describing how the system will function. In our case, the main goal of the dissertation is to demonstrate that by providing an image (live image from video) as an input to the system, it must be capable of detecting the object and recognizing the object’s class. To accomplish this, we must first train the SSD with many input images. These images will be taken out of the datasets and handled in line with the prerequisites for SSD input mentioned earlier. After the training phase is finished, a second phase begins in which the system must output the original image along with the precise bounding boxes and pertinent description surrounding the object outside, giving an input image to the pre-trained model. The objective is to have an interactive testing layer during the live phase to test system-wide metrics like mean average precision. The proposed system architecture and the deep learning methods applied for a real-time object detection system are shown in the figure below.

**FIGURE 7:**
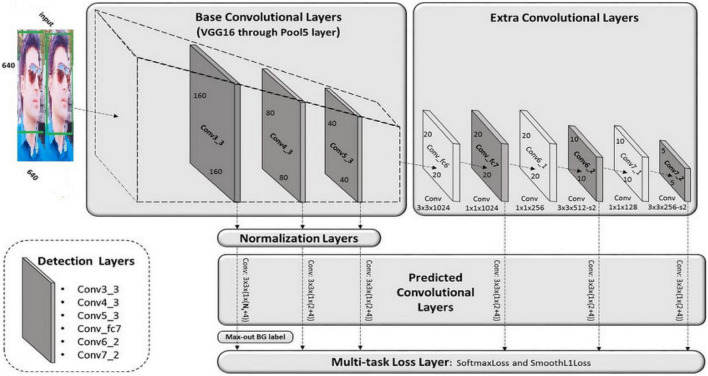
The system architecture.

The SSD algorithm that we will use in our proposed system is depicted in the figure below. We have also shown the datasets that are training and testing data required for the development of our system in the following architecture (Figure 7). We also displayed evaluation metrics such as recall, precision, and accuracy. When the system detects an object image, it goes through several steps, such as taking an image with a web camera, extracting the features, classifying the class of the object, testing with the dataset, running the detection algorithm, and finally displaying the output, such as an image with a bounding box. The diagram above depicts how the system will capture and detect the object.

### Design of data processing module

**FIGURE 8:**
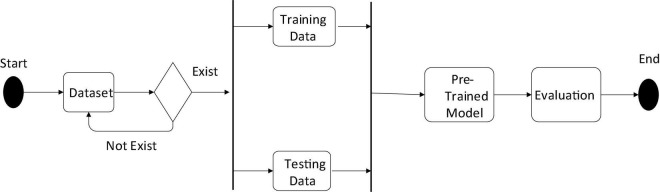
Activity diagram of the data processing module.

This section will go over the data processing procedure. As shown in Figure 8, we must have our dataset because we have divided the dataset into exercise and testing data. When the system starts, it checks the available dataset; if the system finds the dataset, it proceeds to the next step, such as training and test data. If the system does not find the dataset, it will look again; otherwise, an error message will be displayed. As shown in the activity diagram, once the dataset is established, the system will take a small portion of the training data, such as 80%, and the remainder as testing data, such as 20%. The system will then proceed to the pre-trained model and detect the object, followed by a final evaluation.

### Class diagram of the proposed system

Class diagrams depict the connections and source code conditions that exist between classes. A class describes the tactics and components of an object in this case, which is a specific component of a programmed or the unit of code that corresponds to that entity. A Class Diagram is a static representation of an application. A class outline depicts the different types of objects in the framework as well as the different types of connections. A class is used to represent at least one object in object-oriented programming. While each object is composed of a single class, a single class can be used to begin multiple articles. A static chart represents the class outline. It refers to the static view of a framework/application. The class diagram in our system included the base network, which will be used for feature extraction, and the SSD, which will be used to localize and recognize the object class. Image scaling, dataset usage, and object attributes will all be considered here. Our system’s main task is to train the models on the given dataset, so that they can successfully detect and recognize the object in a video sequence.

### System implementation

**FIGURE 9:**
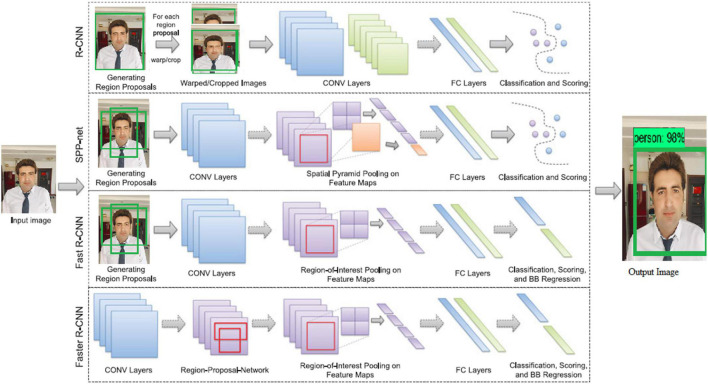
System implementation.

In the data processing module, we will discuss how the data will be processed and how we will practically implement it using Python coding. We will discuss the practical implementation of the data processing module in this section. In this module, we must consider the train and test data, as well as the evaluation, to determine the accuracy. The pre-trained model and SSD will be trained on train data first. When the system boots up, it loads the train data first, followed by the trained model, and then, the test data are passed to the trained model for further evaluation to ensure accuracy. Figure 9 depicts the implementation procedure, demonstrating how it works.

It converts raw detection dataset to TFRecord for object detection. It converts detection dataset to TFRecords with a standard format allowing to use this dataset to train object detectors. The raw dataset can be downloaded from the internet. Detection dataset contains 1,481 training images. Using this code with the default settings will set aside the first 500 images as a validation set. This can be altered using the flags.

#### Implementation of deep learning module

In this module, we will deliberate how to implement the deep learning module and how it will work in our implementation? In deep learning module, we have the basic proposed algorithm which SSD and the pre-trained models, which is, for example, SSD_MobileNet-c1_coco model. In deep learning module implementation, whenever the webcam opened, it will take the image of the object and this image will pass to the training and testing dataset. In the next phase, the pre-trained model will be activated and be prepared and similarly these images and model will pass to the deep learning techniques which is SSD in our case. Similarly, the object will be detected and recognized. The deep learning module for object detection basically includes many steps, which are the dataset processing, the training of the models, and the prediction. Prediction is for the recognition of the objects. The training phase will be coded in this module, which is the model training on the available dataset. Figure 4 represents the flow chart of the deep learning module. The system will take starting step from the input image with the help of the web camera, and then, the further steps will take place.

The deep learning module of our proposed system includes the SSD basic detection algorithm as well as the SSD_MobileNet_v1_coco pre-trained model. In this section, we will write code for the SSD as well as the mobile net, which serves as the base network and is used to extract features.

#### Implementation of static object detection module

We will discuss the static object detection procedure and its implementation in this module. Object detection, as mentioned in the design section, is the process of object recognition (classification), localization (bounding boxes), tracking, and extracting object information. These processes are inextricably linked to object detection. The main goal of the classification stage is to determine the object’s class or recognize what the object is. The class of the object is identified here. Localization is the process of defining the location of an object or objects in an image or localizing an object within the frame. In this module, the image will be taken as input from the web camera and converted to grayscale. Later, cascading will be applied to the image to find out the object. If it is founded successfully, the next phase will be started. Otherwise, it will not proceed. Whenever the object is detected, it will be displayed with a bounding box.

#### Implementation of pre-defined objects module

In this module, we will implement the pre-defined object module that we designed and discussed in the system design section’s pre-defined object detection section. Pre-defined objects are essentially the datasets, as we have already defined the objects (labeled data) and trained the model to select objects from the pre-defined dataset. If the input data are valid, the pre-trained model will be called upon, the image will be compared with the pre-defined object images, and the most nearby and related object images with their names will be displayed. The pre-defined object detection module is responsible for this.

#### Implementation of moving object tracking module

Object tracking in video or live recording is the process of determining the movement and status of an object. The purpose of an object detection system is to categorize and locate (localize) all objects in an image. In our system, the basic input for the detector is an image comprising the object, and the output is a list of the rectangular bounding box. This procedure involves background subtraction techniques. It also includes the processes of localization and classification, just like static object detection and the moving object tracking module. This module is mostly defined for vehicle detection on roads. This type of module is used for traffic purposes. It will detect the moving object, such as a person on the street or on the road, as well as the vehicles that are moving on the road. The system will take an image from the moving object *via* web camera and apply the background subtraction techniques. Similarly, the image from the live stream will be detected and the system will follow the next step.

The system will then proceed to apply the SSD algorithm techniques to the trained dataset as well as the pre-trained models in the following phase, image processing. The next phase is the object recognition phase, in which the object is recognized and the results are displayed. Here, we will use the same code as the static object detection code. We have used two terms like static object detection and moving object detection, we mean that our system is capable of detecting both scenarios’ objects. Some of the systems do not detect the moving objects, but our system can track the moving object, such as the person walking on the street or the vehicle moving on the road, and the system can detect them.

Detecting objects in a moving scene is the first step in video analysis. An object detection mechanism is used when a moving object appears in a video scene. The object detection method relies on information in a single frame. Motion detection is a significant and difficult task when compared to static object detection (Ahmad et al., 2021, 2022). When an object moves, the system takes an image of it and then applies the object detection algorithm, which is the SSD algorithm. But during the tracking phase, the system will also use the feature extraction techniques, which is in our case the mobile net techniques which provides the high-level features for the detection algorithm. Similarly, both algorithm such as the base network mobile net and the detection network SSD will combine detect and track the moving object.

#### Implementation of object recognition module

In this section, we will implement our system’s object recognition module. Essentially, object recognition entails identifying the object class and displaying the object with its class name and bounding boxes. Object recognition consists of several steps, including image input, detection, classification, recognition, and finally the result.

In reality, we have various object classes such as human, car, cup, bottle, and so on. The task at hand is to locate a specific object in an image or even a video sequence. It is a fundamental vision issue because object recognition engines continue to struggle, in contrast to humans, who can detect and identify a wide variety of objects in images or videos that differ in viewpoint, color, size, or even when the object is partially obstructed (Lee et al., 2017). During the implementation phase of the object recognition module, static object recognition is mostly used for cars that have been parked in a non-parking area or an area that is not designated for parking.

Detection and recognition are two distinct concepts. The detection process is the first step toward recognition. Detection is the process of locating semantic objects in a video scene, such as humans, cars, and animals. The object detection algorithm is primarily used to extract the features that can be used to identify the object class. Detection is the detection of something in front of the web camera. The purpose of recognition is to recognize and identify the class of the detected object, which means whether the detected object is a human, a car, or something else.

#### Critical algorithm and pseudo-code

As previously stated, the critical algorithm in our case is SSD. In this section, we will briefly explain the critical algorithm and how we can code and implement it to build a real-time object detection system. This system handles the objects of various sizes by providing highlight maps from various convolutional layers to the classifier. This meta-architecture is faster than others, but it lacks detection precision because it completes everything all at once.

To meet our requirements, we are training the SSD algorithm alongside pre-trained models. On our local laptop, we implement the coding process, and we successfully run the following code with the required results. The SSD layer is built around a feed-forward CNN, which generates a fixed estimate collection of bounding boxes and object class cases that are displayed within those boxes. The input image is passed through several convolutional layers before being examined by the SSD layer. The SSD design is built on the venerable VGG-16 architecture but without the fully connected layers. The VGG-16 network was chosen as the base network due to its excellent performance in tasks requiring the classification of high-quality images and its track record in problems where transfer learning can aid in research advancement.

## Experimental results and evaluation

• Precision               $\text{P}=\frac{\text{TP}}{\text{TP}+\text{FP}}$
• Recall               $\text{R}=\frac{\text{TP}}{\text{TP}+\text{FN}}$
• Accuracy is defined by the following formula $\text{AC}=\frac{\text{TP}}{\text{TP}+\text{FN}+\text{FP}}$
• Mean average precision

$\text{mAP}=\mathrm{Average}\,\mathrm{Precision}\,\left(\frac{\text{TP}}{\text{TP}+\text{FP}}\,\right)$

$\text{mAR}=\mathrm{Average}\,\mathrm{Recall}\,\left(\frac{\text{TP}}{\text{TP}+\text{FN}}\right)$

**FIGURE 10:**
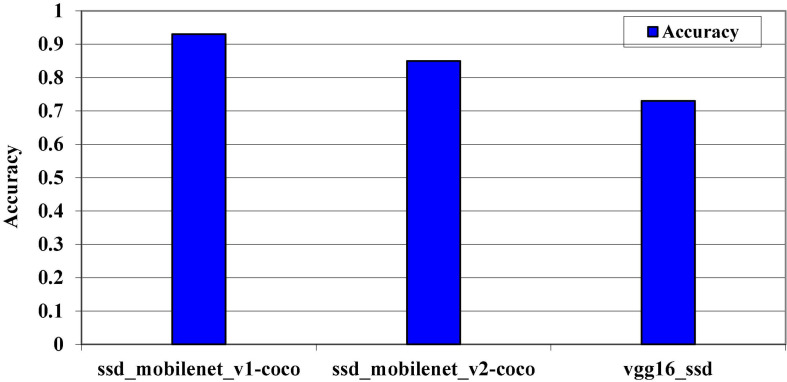
Model’s accuracy levels on MS COCO dataset.

Accuracy refers to exactness or quality, whereas review refers to completeness or quantity. High accuracy means that a calculation returned significantly more relevant results than insignificant ones, whereas high review means that a calculation returned most of the important results, as shown in Figure 10.

**FIGURE 11:**
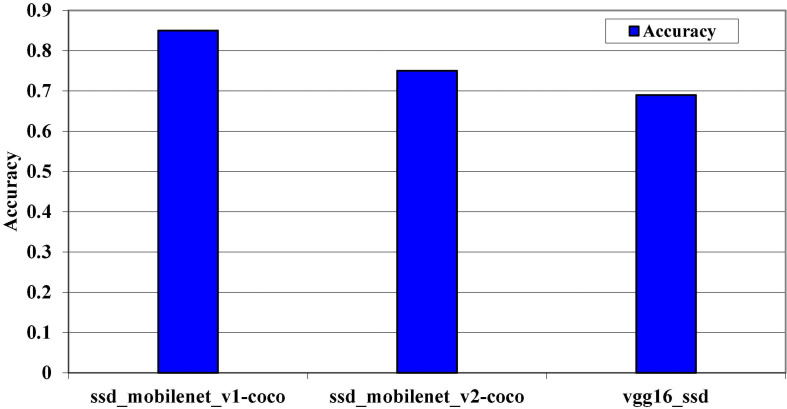
Models prediction error.

**FIGURE 12:**
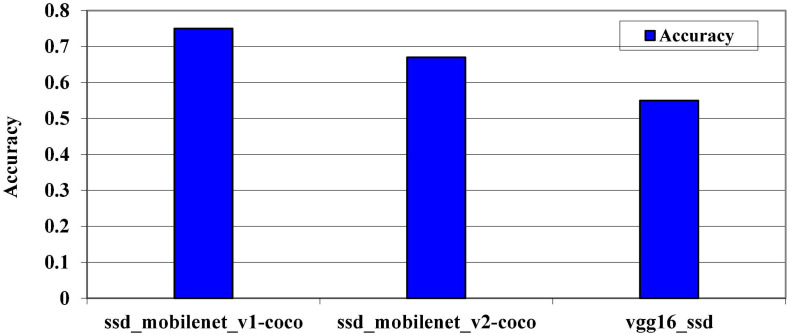
Model’s accuracy levels on kitti dataset.

**FIGURE 13:**
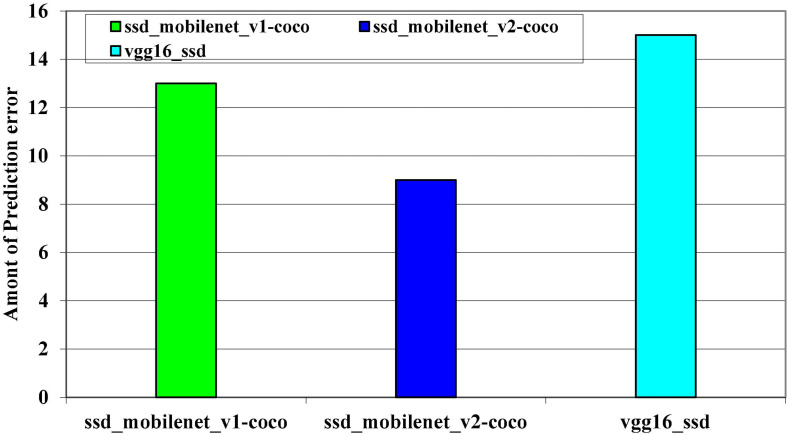
Model’s accuracy levels on Pascal VOC dataset.

Figure 11 clearly shows that the pre-trained model SSD_MobileNet_v1_coco outperforms the other two models on the MS COCO dataset. Although the accuracy level changes as we change the dataset size, this is because the hug size of the dataset can affect the prediction algorithm’s accuracy level. The accuracy level on the kitti dataset and the Pascal VOC dataset is represented by the next two graphs. The Pascal VOC dataset is the largest dataset in our system. The most important thing we noticed during the evaluation phase is that the accuracy increases at different epochs from start to finish, as defined by the 24 epochs, as shown in Figures 12, 13.

The accuracy levels of the pre-trained models on the kitti dataset are depicted in Figure 11. To determine which model have high accuracy on the kitti dataset, all three models were trained on it. On the kitti dataset, the SSD_MobileNet_v1 has high accuracy.

**FIGURE 14:**
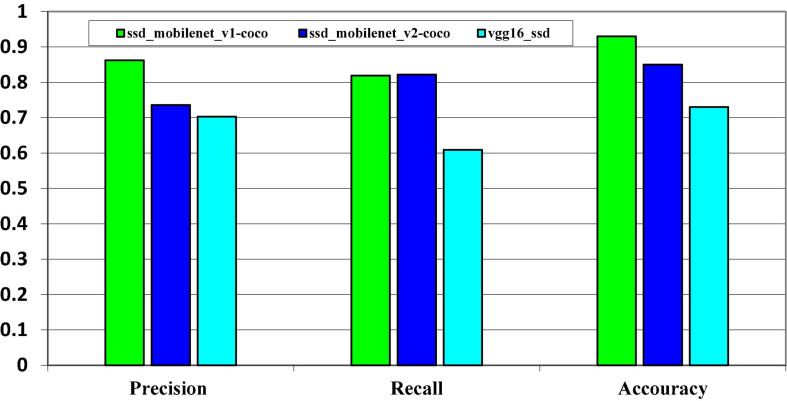
Models evaluation metrics on MS COCO dataset.

The accuracy levels of the pre-trained models on the Pascal VOC dataset are also represented in Figure 13. All three models were trained on the Pascal VOC dataset to determine which models perform well on this dataset. On this dataset, it is discovered that the SSD_Mobilenet_v1 has a high accuracy. Essentially, the Pascal VOC dataset is a very large dataset that contains thousands of images, but in our case, our laptop cannot support such a large dataset, so we must reduce the size of the dataset. In some cases, we will consider at least 1,000 images. The models evaluation metrics on MS COCO dataset is shown in Figure 14.

**FIGURE 15:**
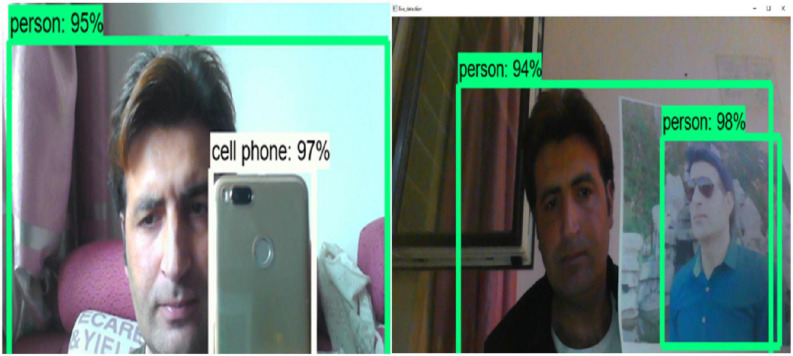
System functionality testing.

The precision-recall cure of the various classes is depicted in Figure 15. As previously stated, we have various class objects such as humans, cars, cups, doors, and so on. Labeling techniques have been used to label these classes and objects with their names. The figure above depicts the precision-recall curve as well as the average precision of each class. The standard precision of a bike, clock, door, drawer, and the socket are much lower than that of other object classes. The trained detector identifies the five most difficult classes in the dataset. The model overlearned the specific time shown on the clock (location of the hour and minute hands) rather than the clock’s structure, which explains the lower accuracy in clock class. There are various types of handles and backgrounds for doors and drawers. The wall’s background color makes it difficult to detect the switch. We compared the performance of the models on the MS COCO dataset in terms of precision, recall, and accuracy. On the COCO dataset, the SSD_MobileNet _v1 has high accuracy.

**FIGURE 16:**
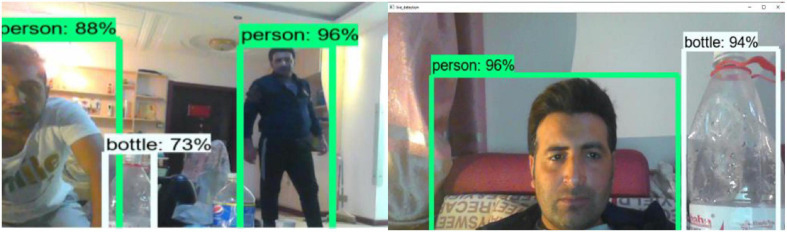
Live objects detection and recognition and accuracy testing.

As illustrated in Figure 16, the number of images on training data has a significant impact on detector performance. The detector’s precision and recall on unseen evaluation datasets improve as the size of the training samples grows. Even though the increase in accuracy is not smooth, we can generalize that detectors trained on more samples have higher accuracies than detectors trained on fewer samples. Because the dataset’s object density is unbalanced, the random split produces unbalanced subsets. The root cause of accuracy fluctuations is an unbalanced distribution of objects on the training dataset.

### System testing

Systems, particularly those involving the internet, must be tested before being made public to address issues. Different problems, such as software or hardware issues, as well as issues such as system security, basic functionality, user accessibility, and adaptability to a wide range of the desktop, device, and operating systems, should be tested and evaluated. The following are the main goals and objectives of the testing phase:

1. The system should be tested to ensure that it meets the requirements for design and development.
2. The system will be tested to ensure that it responds correctly to all types of inputs.
3. Perform its functions accurately and within a reasonable time frame.
4. Installed and used in the intended environments.
5. Obtain the overall desired and pre-defined results.

#### System functionality testing

System functionality testing entails testing the system’s primary function. This section looks at the overall framework’s utility. From the smallest module to the entire framework module. We examine whether they work as expected and whether they can do the job for which they were designed. The system’s capabilities are built on various modules. In any case, all modules collaborate to carry out the object detection system. Figure 15 depicts the functionality of our system, which is fully functional and operational. The main goal of system functionality testing is to see the system’s functions in action and to observe the system’s functionality results in action. The system functionality testing yielded the desired result.

The figures above depict the overall functionality testing of the system. It clearly demonstrates that our system is fully operational and functional. The percentage represents the detection and recognition accuracy level.

#### Module testing

Modules are distinct parts of a framework that can eventually be coordinated to create a fully functional framework. We have a deep learning module, a static object detection module, a moving object tracking module, a pre-defined objects module, and an object recognition module in our system (Hoang Ngan Le et al., 2016; Han et al., 2017). Following implementation, we thoroughly tested and ran these modules to ensure that no bugs were discovered. These modules function properly. If any of the modules contained bugs or programming errors, the system would not function properly and would not be operational.

#### System performance and accuracy testing on live detection

**FIGURE 17:**
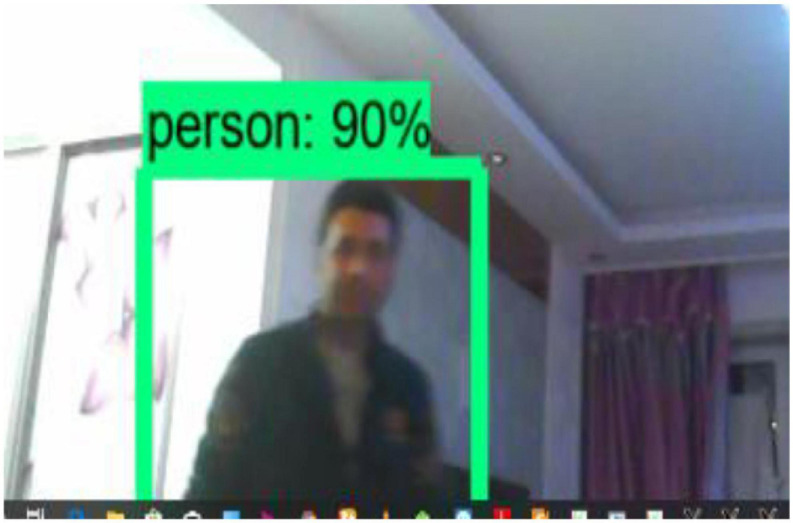
Moving object tracking.

As previously stated, our system performs better in live application testing. One of the requirements was that the system be operational on a local PC. We ran the system through its paces on our laptop. The system successfully detected various objects and can identify the class of the detected objects. The bounding boxes are used to separate the objects. The boxes can be used to locate an object in a video sequence. Figure 17 is a demonstration of live testing. Because the object is not moving in this type of static object detection module testing, the system successfully detected the objects and recognized the detected object classes (Shafiq et al., 2020; Shafiq and Gu, 2022; Wahab et al., 2022).

Figure 16 depicts system functionality on a live stream. Each detected object has an accuracy associated with it. The bounding boxes are used to separate the objects. The moving object tracking functionality is depicted in the figure below.

The object in Figure 17 is not static, but rather continues to move; this is an example of a moving object tracking module. The system detected the moving object and identified the detected moving object class. The class name is displayed in a green color box, along with the accuracy level.

## Conclusion

The primary goals of this research were to investigate deep learning and its various techniques and structures, followed by the development of a real-time object detection system that uses deep learning and neural systems for object detection and recognition. Similarly, the system had to be kept running on reasonable equipment. Several deep learning structures were tried and evaluated during the coding procedure. The main contribution of this paper is to test the pre-trained models with SSD on various types of datasets to determine which model is more accurate in detecting and recognizing the object, as well as which model performs best on which dataset. As a result, on the MS COCO dataset, we concluded that the pre-trained model SSD_MobileNet_v1_coco outperformed the others.

We achieved good results and discovered that we had designed and developed a real-time object detection system successfully. During the system testing phase, we also test the various modules of our proposed system and the detection accuracy results. We also assessed the system’s functionality. Graphs have been used to represent the evaluation results. We also tested the dataset with pre-trained models to see which models have high accuracy under different conditions.

This work can also be extended to detect the action of the objects, such as detecting what the object (person) is doing and whether the person is using a mobile phone or a laptop. In other words, the system should act intelligently in order to detect the action of a specific object. If the person is driving, the system should detect and display this information. It will also be very interesting to expand this system to track the movement of vehicles on the road. The velocity of the moving object will be calculated for this purpose using some type of programming, and the output will be displayed on the screen. On the other hand, the CCTV camera should be programmed, so that it can use this system to calculate the motion (velocity) of moving vehicles on the road.

## Data availability statement

The raw data supporting the conclusions of this article will be made available by the authors, without undue reservation.

## Ethics statement

The studies involving human participants were reviewed and approved by the National Research Foundation of Korea (NRF) grant funded by the Korean Government (MSIT) (No. NRF-2021R1F1A1062181). The ethics committee waived the requirement of written informed consent for participation. Written informed consent was obtained from the individual(s) for the publication of any potentially identifiable images or data included in this article.

## Author contributions

FW: conceptualization and investigation. FW and IU: methodology and writing—original draft preparation. FW and AS: software. FW, IU, and RK: validation. FW, IU, and AC: formal analysis. IU and MA: resources. FW, IU, RK, AC, and MA: data curation. IU and AS: writing—review and editing. IU and RK: visualization. IU and AC: supervision. IU, AC, and MA: project administration. AC: funding acquisition. All authors have read and agreed to the published version of the manuscript.
